# ‘It’s not good to eat a candy in a wrapper’: male students’ perspectives on condom use and concurrent sexual partnerships in the eastern Democratic Republic of Congo

**DOI:** 10.1080/17290376.2018.1516160

**Published:** 2018-08-27

**Authors:** Maroyi Mulumeoderhwa

**Affiliations:** Faculty of Top Management, Institute for Reconciliation and Social Justice, University of the Free State, Bloemfontein, South Africa

**Keywords:** Condoms, concurrent sexual partnerships, girlfriend, young men, HIV/AIDS

## Abstract

This paper reports on fieldwork carried out in 2011 with aim to investigate young men’s perspectives about condoms use, concurrent sexual partnerships and sex in the context of HIV/AIDS. This study employed a qualitative approach to collect data from 28 boys aged 16–20 from two urban and two rural high schools in South Kivu province. Four focus group discussions and 20 individual interviews were conducted among them. The findings showed that most students identified condoms as unsafe and untrustworthy. Reasons given for the mistrust of condoms were related to the belief that condoms do not give enough protection from Sexually Transmitted Infections, HIV and pregnancies. Most participants believe that condoms have a ‘small hole’ or are unreliable and are therefore not effective in prevention. They also mentioned that condoms encourage inappropriate sexual activity. They prefer flesh-to-flesh sex rather than protected sex using a condom. However, a few participants acknowledged the importance of condom use. Despite the risk of HIV transmission, boys believe that it is appropriate for them to have concurrent sexual partnerships. They justified the concurrent sexual partnerships as a way of ensuring that they cannot miss a girl to satisfy their sexual desire. Given the boys’ failure to use condoms and their strong inclination to concurrent sexual partnerships, there is a need for heath groups and stakeholders within the area to increase awareness about condoms’ effectiveness and improve knowledge dissemination on Sexually Transmitted Diseases and how they are prevented.

## Introduction

Condoms are strongly mistrusted and are based on the perceived lack of protective efficacy (Maticka-Tyndale, 2012). These include beliefs that condoms do not fully protect against STIs or HIV (because they slip, break or allow infectious agents to pass through) or even that condoms increase the risk of HIV transmission (because they are fabricated with the virus in them or with holes in them as a plot to exterminate the people of sub-Saharan Africa, or that the virus is small enough to pass through condoms) (Maticka-Tyndale, 2012, p. 63). Similarly, several studies in Nigeria (Okonkwo, 2010), South Africa (MacPhail & Campbell, 2001), Malawi (Chimbiri, 2007), Kenya (Maticka-Tyndale & Kyeremeh, 2010; Maticka-Tyndale et al., 2005), Democratic Republic of the Congo (DRC) (Bosmans, Cikuru, Claeys, & Temmerman, 2006; Kandala, Lukumu, Mantempa, Kandala, & Chirwa, 2014; Kayembe et al., 2008a; Kidman, Palermo, & Bertrand, 2015) found that participants believe condoms cannot prevent infection. Such a belief can present a major impediment to condom use.

## Condom inconsistency and justification

Various studies conducted in sub Saharan Africa maintain that condoms are associated with promiscuity, unfaithfulness and distrust (Bosmans et al., 2006; Clark, Bruce, & Dude, 2006; Hallman, 2004; Maticka-Tyndale & Kyeremeh, 2010; Montgomery et al., 2008; Plummer et al., 2006; Tavory & Swidler, 2009). For these reasons, condoms are not only considered useless, but are also viewed as a destructive factor in a relationship built on trust, especially in marriage (Brown, 2015; Chimbiri, 2007; Ghosh & Kalipeni, 2005; Maharaj & Cleland, 2004). In a study conducted among university students in Goma, participants believed that condom use can reduce pleasure. 55% of participants stated that condoms can tear (Masoda & Govender, 2013).

Many of the adolescents, from the study conducted in Kinshasa and Bukavu, doubted the reliability of condoms, and girls who would use them were regarded as sexual easy givers (Bosmans et al., 2006). A number of studies in the DRC find that regular condom use depends on a range of factors, including the knowledge that condoms are ineffective in preventing infection with HIV (Carlos et al., 2015; Kayembe et al., 2008a, 2008b; Kidman et al., 2015; Van Rossem, Meekers, & Akinyemi, 2001). In another study in Kinshasa and Bukavu participants indicated that condoms did not offer full protection (Bosmans et al., 2006). A study conducted in South Kivu finds that social constructions of masculinity not only actively encourage the idea that ‘men cannot control their sexual urges’, but also hinder women's negotiation ability to negotiate safe sex and refuse it (Mulumeoderhwa & Harris, 2013).

## Condoms supply limitations

A Survey conducted in the DRC indicates that only 20% of Congolese women from 15 to 49 have used contraceptive methods (31% in cities and 15% in villages) (Congo Democratic Republic DHS, 2013–2014). This may actually result from the lack of investment and maintenance of public health services, combined with a massive withdrawal of multilateral and bilateral cooperation in 1992 and on-going war since 1996, resulted in the collapse of the health system of the DRC, which was left solely in the hands of non-governmental organisations (NGOs), churches and private assistance (Van Herp, Parqué, Rackley, & Ford, 2003). In Bukavu, for example, the decision not delivering condoms is in accordance with the policy of Catholic Church. Those who have responsibility for the peer education programme and Health Department of the diocese fiercely refuse to supply condoms, particularly to girls living on the street and surviving as sex workers. They also do not consider condoms as a means of protecting them from unwanted pregnancies, HIV or other sexually transmitted infections, but rather as a persuasion to continue their way of life (Bosmans et al., 2006). Sociocultural barriers and strict conformity to religious doctrine can prevent adolescents from receiving appropriate and comprehensive sexuality education and cause peer education officers to block condom supplies (Bosmans et al., 2006, p. 8; Parker et al., 2013). In fact, close collaboration between religious leaders and HIV prevention programmes is necessary to ensure success (Carlos et al., 2015; Muanda, Ndongo, Messina, & Bertrand, 2017; Parker et al., 2013).

## Multiple partnerships

Although condom has been a main factor explored in the literature, several other factors are hypothesised to influence effective prevention of HIV and Sexually Transmitted Infections. A study using cluster sampling conducted in the eleven provinces of the Democratic Republic of the Congo among 8617 adolescents, indicates that adolescents reported having at least two sexual partners; males had more partners (62.2%) than females (35.3%) (Kayembe et al., 2008a). Another study conducted in Kinshasa also finds that most participants were already sexually experienced, and have had concurrent sexual partners while not using condoms consistently. The low rate of condom use and the high percentage of young people reporting concurrent sexual partners suggest that Congolese teenagers and young adults remain a group highly at risk of STIs and HIV transmission (Kayembe et al., 2008c). Concurrent sexual partners and inconsistent condom use can seriously hinder the effective fight against HIV/AIDS (Carlos et al., 2017; Lusey, Sebastian, Christianson, Dahlgren, & Edin, 2014). Thus, concurrent sexual partnerships can increase an individual’s risk for acquiring HIV after the inconstancy of condom use (Carlos et al., 2017; Steffenson, Pettifor, Seage, Rees, & Cleary, 2011; Tarkang, 2015). The assumptions mentioned above may also hinder the prevention of HIV. To our knowledge, this is the first study in South Kivu that has investigated the attitudes on condoms, concurrent sexual partnerships and the context in which adolescent sexual activity is negotiated. Drawing on qualitative data, this study takes into consideration the sociocultural context in which personal and collective experiences, knowledge, attitudes and behaviours are constructed and negotiated. The aim of this study is to investigate young men’s perspectives about condoms, concurrent sexual partners and sex in the context of HIV/AIDS.

## Studying adolescents

Given that it is important to investigate the attitudes of men towards condoms and concurrent sexual partnerships, actually, considering men in projects on violence against women is important and enables to reduce ‘problems brought by immoderate manhood and harmful concepts of masculinity’ (Adomako & Boateng, 2007). The UNFPA (2006, p. 5) similarly maintains that reducing gender-based violence would benefit from the support and involvement of males since ‘men are increasingly adopting notions of “masculinity” that restrain their humanity, and put their partners and themselves at risk’. This study focuses on adolescent young men, and it is justified by the realisation that the process of becoming an adolescent, as experienced by them is, in most cases, characterised by the differentiation of behaviours and controls that impinge differently on men. Adolescents provide a perspective on how gender norms are socialised and a sense of how early such socialisation occurs. Adolescents are in the liminal position of not quite children or adults. They should be approached because they are old enough and able to explain how chronicle events, including violence that is pertinent in their lives.

## Theoretical framework

The present study was guided by gender and power theory. Gender and power theory, which focuses on the social norms associated with relationships between men and women, may inform our understanding of the use of condoms and concurrent sexual partners (Connell, 1987). Some scholars extended Connell’s theory into public health to include behavioural and biological risk factors as explanations for women’s increased risk for HIV. Their model includes alcohol and drug use and high-risk concurrent sexual partners who have been linked to sexual intercourse (Bal, Mitra, Mallick, Chakraborti, & Sarkar, 2010; Versteeg & Murray, 2008). Others DePadilla, Windle, Wingood, Cooper, and DiClemente (2011) validated Wingood and DiClemente’s model with empirical data demonstrating the relationship between theoretical constructs of gender and power and condom use (Wingood & DiClemente, 2000). Pulerwitz, Amaro, De Jong, Gort maker, and Rudd (2002) found that in the construct of sexual relationship, power may account for failure in the use of condoms.

Additionally, gender and power theory outlines norms governing social and sexual relationships between men and women (Connell, 1987). It provides a practical tool for explaining how gender norms and beliefs are associated with HIV risk and sexual behaviour (DePadilla et al., 2011; Hahm, Lee, Rough, & Strathdee, 2012; Teitelman, Tennille, Bohinski, Jemmott, & Jemmott, 2011; Weine et al., 2013; Wingood & DiClemente, 2000). Gender and power theory establishes that women’s limited economic position and dependence on men for financial support (Connell, 1987; Wingood & DiClemente, 2000), diminishing their ability to negotiate safer sex or condom use (Hardee, Gay, Croce-Galis, & Peltz, 2014; Wingood & DiClemente, 1998). Women’s lack of negotiating power within sexual relationships due to male control, power or coercion (Connell, 1987; Wingood & DiClemente, 2000), may put women in a more vulnerable position when it comes to HIV risk (Ackermann & De Klerk, 2002; Jewkes & Morrell, 2012; Raiford, Seth, & DiClemente, 2013; Riley & Baah-Odoom, 2012; VanderDrift, Agnew, Harvey, & Warren, 2013). The broader social principles governing gender norms and expectations often define sexual behaviour for women and outline normal female behaviour within relationships (Connell, 1987; Wingood & DiClemente, 2000).Thus, gender norms may lead to the expectation that women are ‘passive’ receivers in sexual relationships, that women might not or should not carry condoms, or expectations that women should defer to men in making decisions about sexual behaviour (Connell, 1987; Wingood & DiClemente, 2000), which leads to greater HIV risk for women (De Santis & Patsdaughter, 2014; Hardee et al., 2014; Horton & Dworkin, 2013; Richardson et al., 2014). This study posits that gender and power may play a significant role in men’s dominance over and oppression of women, thus exposing women to Sexually Transmitted infections and HIV.

## Method

A qualitative research method was employed to investigate young men’s perspectives about condoms, concurrent sexual partnerships and sex in the context of HIV/AIDS. The study has also employed grounded theory, one of the qualitative approaches. Qualitative research is an examination process of understanding based on distinct methodological traditions of investigation that explore a social or human problem. The researcher builds a complex, holistic picture, analyses words, reports detailed views of participants, and conducts the study in a natural setting (Creswell, 1998, p. 15). One of the features of qualitative research is that it involves systematic discovery. Its objective is to produce knowledge of social events by understanding their meaning to people, investigating and documenting how people exchange ideas with each other and how they understand and interact with the world around them (Ulin, Robinson, Tolley, & McNeill, 2002, p. 26). By employing this method, we were able to understand participants’ attitudes and behaviours. In fact, the qualitative researcher needs to attentively watch incidents and actions as they occur without any action affecting them or interference (Babbie & Mouton, 2001, p. 270).

This study employed two of the qualitative methods for data collection which are namely: focus groups and individual interviews. Focus groups and individual interviews were conducted with boys. The research assistant and I conducted focus groups and individual interviews. In a focus group, we played the part of being a guide, but within the group, a multitude of interpersonal dynamics occurred and it was through this group interaction that data were generated (Morgan, 1993). We played a low-key role and gradually introduced a limited number of questions at appropriate times. For example, one focus-group question asked whether they thought it was it important to use condoms. If yes/not explain why. The related interview question was ‘If your girlfriend or wife asked you to use a condom, what would you think?’ Other questions included in our research were: (1) Is it OK to have more than one girlfriend at a time? If yes/not explain why. (2) How many girlfriends do you have at the present time? (3) Can you have a relationship (with your girlfriend) without sex? If yes/not explain why. Focus groups can help participants develop a point of view that goes beyond their individual context and thus may change ‘personal troubles’ into ‘public issues’. The group process can also nurture collective identity and provide a point of contact to initiate grassroots transformation. Although focus groups can, in theory, simply reflect or monitor change, there is always the potential for the focus group process itself to initiate changes in respondents’ thinking or understanding, through exposure to the interactive process (Barbour, 1999). Focus groups can bring significant support to sensitive research. They can be useful in enabling access to particularly sensitive research populations and giving voice to sections of the community that frequently remain unheard (Farquhar, 1999). We chose focus group discussions as a key tool of data collection because of our interest of understanding of attitudes and behaviour as a social phenomenon which are strongly influenced by peer norms. The individual interview was chosen as a supplementary technique in this study to get opinions that could be difficult to divulge in focus group discussions.

Four focus group sessions were conducted in Swahili, the language spoken by the participants, and lasted between 90 and 120 minutes. The interviews took place during the two weeks that followed each focus group, and lasted approximately 60 minutes. This approach was vital to gaining access to the perspectives of participants who could be ostracised by their peers because of their views or become extremely passive in the focus groups. The interviews also enabled us to personalise the issues raised in the focus groups, to probe responses where relevant, and helped to explore further and confirm themes emerging from the focus group interviews. That being said, the specific benefit of focus groups derives from the interaction, discussion and exchange of ideas among the participants. The use of direct quotations from participants is a way of guarding against a dominating individual, as was following up in the interviews. I transcribed the verbatim audio from the tape recorder from Swahili to English for later analysis. Data were translated, reread and back-translated from Swahili to English.

## Participants

Fieldwork and data collection were carried out in 2011 in four secondary schools in Bukavu and Kavumu. Bukavu, the capital of South Kivu province, is overcrowded with people who have moved from elsewhere seeking greater security. The population was about 800,000 in 2012. Kavumu is a rural area located some 50 km to the north of Bukavu. Given the widespread destruction and deterioration of basic infrastructure throughout the province, people in Kavumu have difficulty in accessing basic social services such as education, sanitary drinking water, and primary health care. Such services are somewhat better in towns, but are limited in both quantity and quality. Most of Kavumu’s houses are built with flat timber and others with mud and timber. In terms of sampling, the four schools were chosen for reasons of convenience but are broadly typical of high schools in the province.

A total of 28 young men participated in the study. The 28 participants (the number was chosen to allow one focus group of seven boys at each school) comprised about a quarter of the students at the schools who were in their final two years of study and were between 16 and 20 years old. The school’s principal and one teacher, from each school of our data collection, chose from grades 11 and 12 the male students who participated in the study. In choosing a sampling, the goal is to select a group of participants who are purposefully located to express themselves on the topic under exploration (Gerson & Horwitz, 2002). Five participants from each focus group were invited to voluntarily participate in individual interviews. The five out of seven focus group participants were chosen for the reason of convenience and that in individual interviews, we may have 20 boys. These individual interviews were conducted with 20 volunteers from the 28 focus group participants. The majority of participants in this study were from the Bashi ethnic group.

## Data analysis

Data from focus groups and individual interviews was analysed using thematic and grounded theory analysis to identify the themes that emerged. This method faces the risk that a researcher may deliberately or unintentionally inject her/his own biases into the results and reach inaccurate conclusions; that is, it may fail in terms of internal validity. Careful action was taken in order to prevent this from happening by having two people to carry out the focus groups and interviews, and two other people to help with the recording and scribing. We drew attention to certain information in description (Huberman & Miles, 1994). We read the entire data in depth in search of meanings and patterns, and wrote down ideas, reflective notes (Creswell, 1998; Huberman & Miles, 1994), and formed initial codes during the analysis process. We engaged in axial coding–causing condition, context, and consequences. We also engaged in open coding–categories, properties, and dimensionalise properties (Bodgan & Biklen, 1992), made contrasts and comparisons (Huberman & Miles, 1994), and developed coding categories. We developed a guide for coding the focus groups and interviews, and reread to identify themes as they emerged. During the coding process, we retained the individual extracts of data and the dominant stories (Braun & Clarke, 2006). We reduced information by sorting material into categories (Bodgan & Biklen, 1992), and noted patterns and themes. We related to categories by building a logical chain of information (Huberman & Miles, 1994). By interpreting data, we engaged in selective coding, development of stories, and developed a conditional matrix. Finally, we presented a visual model and propositions. We aimed at reporting experiences and relying on voices and interpretations of participants through extensive quotes, presented themes that represent words used by participants, and advance evidence of different views on each theme (Creswell, 1998, p. 76). Vicsek (2007) suggests often citing in thematic analyses not only isolated manifestations, but also fragments of discussions containing several contributions. In the process of preparing this article, we had a number of discussions concerning the codes that emerged under each theme. The themes we identified arose largely from the data that we collected through questions asked during the focus groups and individual interviews, which, in turn, arose from our research objectives. The views expressed in this study were very similar between both rural and urban respondents. There were strong similarities in views expressed in the focus groups and interviews and only slight contradictions were observed in individual interviews where some participants disagreed with the dominant views from the focus groups. Participants, from urban area, indicated that the women’s initiative to suggest condoms should not be perceived as a sign of having sexual relations with a number of partners.

## Ethical considerations

All procedures necessary in studies involving human participants were performed in accordance with the ethical standards of the institutional and/or national research committee and with the 1964 Helsinki declaration and its later amendments or comparable ethical standards. Ethical clearance for this study was granted by University of KwaZulu-Natal’s Ethics Committee and permission was also obtained from the South Kivu Department of Education and from the schools where the study was conducted. Research procedures were explained to participants, stressing that their participation is voluntary. No one refused to participate. Contrary to that, participants were very engaged during the focus groups and interviews. Informed written consent was obtained from all individual participants, and parental permission was obtained for participants under the age of 18. Confidentiality and anonymity were strictly respected, and pseudonyms were used in order to identify the responses. This study reports the respondents’ comments in their own words and the quotations presented are, unless otherwise noted, representative of the beliefs articulated by the majority of participants.

## Results

This study investigated young men’s perspectives about condoms, concurrent sexual partners and sex in the context of HIV/AIDS. The following themes and sub-themes emerged from the study: ‘you cannot eat a candy in a wrapper’, condoms convey ‘have sex and satisfy your sexual urge’, condoms are ineffective, a girl who suggests a condom is untrustworthy, and concurrent sexual partnerships help make a choice of spouse.

## ‘You cannot eat a candy in a wrapper’

Young men socially construct their ideal of true love in unprotected or ‘flesh-to-flesh’ sex. Most participants, during focus group discussions, indicated that young men prefer flesh-to-flesh sex rather than protected sex using a condom:

… Nowadays’ youth often say: ‘you cannot eat a candy in a wrapper, which means skin-to-skin’. It is not any youth who agrees to use condoms. (Bahati, 18, urban boy)

There are boys who say that they cannot eat a candy in a wrapper, and that they must have flesh-to-flesh sex. Such boys may think that a girl does not love him if she suggests him to use condom. (Iste, 18, rural boy)

Most participants had a strong preference for ‘flesh-to-flesh’ sex because condoms were perceived to reduce sexual pleasure. Therefore, they promote beliefs such as ‘you cannot eat a candy in a wrapper’. This adage is seen as a motive to promote unsafe sex. Boys’ preference for sex without condoms may be one of the contributing factors to the spread of Sexually Transmitted Infections and HIV.

## Condoms convey ‘have sex and satisfy your sexual urge’

Most participants perceive condoms to encourage inappropriate sexual activity. In effect, they believe that condom promotion campaign and sell entail young people to have sex:

The fact of selling condoms encourages the widespread of sexual violence. It is as if people are conveying a massage saying: ‘have sex and satisfy your sexual urge’. This can discourage men to hold their sexual urge until they get married, because they know that they cannot get diseases. (Gustave, 18, rural boy)

I think that condoms encourage people to become sexually active. Condoms convey the message to people that they cannot get HIV or pregnancy. This has become a vehicle to encourage sex. (Leopold, 20, rural boy)

I would like to say that condom is bad because it encourages sexually activities in the country. People are using condoms because they know that the condom protects, that is why we advise those who make condoms to reduce their production or stop making them. (Clever, 17, rural boy)

Most participants believe that the commerce of condoms encourages sexual activities in the community. Thus, they strongly suggest the cessation of their production. For boys, the selling and supplying of condoms portray a message that invites men to have sex. Condoms are not only difficult to access privately but there are also problems to negotiate their use as women lack the power to do so.

For some participants, the suggestion of condom use should not be mentioned in the household setting. Boys’ beliefs are actually linked to religious practices as they emphasise that the Bible forbids men to pour sperm on the ground.

For married men the condom is not an option, they are not allowed to use it, because the Bible forbids men to pour sperm down [everybody laughs]. (Clever, 17, rural boy)

Most Christian churches in the country discourage the use of condoms. They instead promote abstinence and monogamy as ways of preventing HIV/AIDS.

## Condoms are ineffective

There is a widespread belief that condoms have a ‘small hole’ or are unreliable and are therefore not effective in preventing pregnancy, HIV and Sexually Transmitted Infections:

It is not good to use condoms, because a condom has a small hole that is invisible to the eyes. Once you use it, you should know that you put yourself in risk of getting HIV. Because disease can enter through that hole, particularly when you ejaculate the sperm passes through it and enters a woman. That’s why, I say that condoms can cause unwanted pregnancy; because once you use it [condom], you expose yourself to danger. (Aristote, 17, urban boy)

It is not ok to use condoms. It seems that research has revealed that there is a small hole; I forgot the name that scientists call it. First of all, the person who uses condoms, if we look among youth, he is not confident of himself. (Bisimwa, 17, urban boy)

… Although the condom has small holes there are other sicknesses that it can prevent. I insist that it is bad to use condoms because they are made by the machine. Therefore, it is possible for condoms to have holes that are invisible. (Baraka, 17, urban boy)

Most participants believe that when people use condoms they deliberately put themselves at risk of contracting Sexually Transmitted Infections as condoms have small invisible holes. Therefore, condoms can only prevent some diseases. Condoms are further described as ineffective because humans make them. Participants also indicate that people who use condoms lack confidence. This clearly demonstrates how immoderate masculinity can put young people at risk of contracting HIV and Sexually Transmitted Infection.

The condom’s ineffectiveness may also be attributed to its fabric. Therefore, young men think condoms can easily break during sex:

I think that it is useless to use condoms because you can wear condom yet you still get disease or get pregnant. A condom is made with a soft tissue that is not strong, when the skin scratches it, the condom can break. Concerning sexually transmitted diseases, it is difficult to protect from them, because I realized that even if you use thousand condoms at once, the way that they are built can cause cancer. You can get sicknesses or pregnancy though you wear them, they can prevent absolutely nothing. (Bahati, 18, urban boy)

The widespread distrust of condoms causes young men to believe that condoms can cause cancer due to the way they are built. Participants might have referred to substances containing condoms.

A few participants believe that the condom can slip off into the vagina during sexual intercourse:

It is not good to use condom because you may wrongly wear it, and therefore stuck in the vagina, and causes trouble. (James, 18, rural boy)

## A girl who suggests a condom is untrustworthy

In response to a question about their use of condoms, most of the participants were clear that the decision to use condoms was a male prerogative. That is, females cannot initiate the condom use. During individual interviews, the majority of young men commented that if their girlfriend or wife asked them to use a condom, they would interpret it as a lack of trust in them or would suspect her of being unfaithful:

If my fiancé or my girlfriend asks me to use condom whereas we always have sex without the condom, I can really doubt of her. I am going to think that she cheated on me. (Espoir, 19, urban boy)

Let’s suppose that your girlfriend suggests you to use condom, for me it is not ok. I can think that she is sick or she avoids falling pregnant. (Aristote, 17, urban boy)

If I were married then my wife insists on using condom whereas I do not like, I may suspect her to hide something that she does not like me to know. I can really investigate to know what is going on. (Toussin, 18, rural boy)

The majority of participants interpret their female partners’ request to use a condom as an implication of their partners having sexual relations with other men outside of the relationship. There is actually a certain stigma that is associated with women’s suggestion to use condoms, as this signifies either infidelity or experience in initiating sex, both often considered to be unacceptable characteristics for women.

Other boys, albeit a minority, spoke in favour of condom use. According to them, women’s request to use condom should not be perceived as a sign of having had sexual relations with a number of partners:

The women’s initiative to suggest condoms should not be considered as if she had sexual relations with a number of partners. On the contrary, young people should be encouraged to carry condoms because there are cases that happen when you are not expected. For example, you are a girl walking in the bush or during the night, you may meet men who rape you but if you have got condoms you may plead with them and suggest them to use them so that you may not get diseases or get pregnant of a child who will not know his father. (Baraka, 17, urban boy)

… Some young people like to drink beer, we know that there are some beers you drink and arouse your sexual urge. Maybe you go with your girlfriend to the nightclub. Although you do not like to have sex with her you get it [urge] once you are under the influence of alcohol. That is why we must carry condoms wherever we go to protect ourselves against STIs and HIV. We have to carry them [condoms], and consider them as weapons. (Issa, 19, urban boy)

Some participants resisted the perception that viewed women requesting condom use as the practice of having sexual relations with a number of partners. During individual interviews, they acknowledge the importance of condoms, and advise their peers to use them in order to prevent Sexually Transmitted Infections, HIV and pregnancies. However, girls’ agency in sexual negotiation may seem difficult. They find it extremely difficult to bargain the use of condoms, especially when, at the same time, they are expected to show a lack of experience in sexual matters.

## Concurrent sexual partnerships as an acceptable norm before marriage

Most participants are very clear that it is appropriate for them to have more than one girlfriend. They will then–by being able to compare girls–be more able to make a good choice of a marriage partner. There is at least some implication that, when he marries, he will be faithful to his wife.

It is ok to have two girlfriends. It is a must to have two girlfriends because you are going to compare them. Meanwhile, you can ask yourself: ‘Is this or that other one who deserves to be married?’ I think having many girlfriends depends on every individual, and according to what he looks for. (Toussin, 18, rural boy)

Boys must have many girlfriends because it is during youth period that you plan your future life so that you may say at end ‘my wife is like other women.’ If you have dated many girls, you must have discovered that women are all the same because you marry once and live with one wife. (Patrick, 17, rural boy)

An eighteen years old boy cannot only have one girlfriend. He must examine girls’ characters because girls are hypocrites. That is why he has to make an effort to have at least two girlfriends before he gets married, he must examine their characters to choose one. (John, 17, rural boy)

The concurrent sexual partnership practice is seen as a weighing mechanism that facilitates a boy’s choosing a spouse among his girlfriends. Most participants base the choice of a spouse on a comparative paradigm. Young men especially avoid marrying girls who easily accept sex or have sex with other men. The spouse’s choice is actually based on her reputation. Through the expression ‘my wife is like other women’, boys convey that a young man who has had more than one sexual partner will remain faithful to his wife as a result of his previous sexual activities and understanding. He may have acquired the sense of contentment, and therefore finds no reason to have sex outside his marriage.

## Resorting to the second girlfriend for sexual gratification

Not all boys’ girlfriends, it should be noted, may be sexual partners. During the individual interviews, when the interviewer asked participants about the number of girlfriends they had, their answers included the following:

It is ok to have many girlfriends because in these days boys are more focused on sex. Now do you imagine if somebody is looking for sex, and does not find it? He has a girlfriend who does not like it. Then, he is obliged to look for another girl who can gratify his sexual urge. (Josi, 18, urban boy)

Some participants view concurrent sexual partnerships as a middle ground strategy implying that the boy eventually keeps a girlfriend just in case his primary girlfriend ‘breaks his heart’. They view the necessity of such a practice for desire for sexual activity. They believe that it is irrational for the man to have only one girlfriend because if she does not agree to have sex, he may have no one to provide it. Sex is viewed as the substance around which a relationship generates its subsistence. Some participants also see benefit in having one girl for a trusting relationship and another one essentially for sex. There is an implication that a boy may have several girlfriends but in the event marry someone else:

We must have two girlfriends at the same time … You must have one that you trust and know that she cannot deceive you, and the other one to have sex with her or to speak unnecessary things with her. (Aimer, 19, rural boy)

A few participants, during the individual interviews, contest the idea of having more than two girlfriends:

It is not ok to have more than two girlfriends … You surely encounter financial problem because nowadays’ girls like boys to take them out. When your girlfriend tells you so, you are obliged to take her out … and you find spending money to all these girls. (Derrick, 19, urban boy)

In fact, it is not good to have more than two girlfriends, but it happens that when you are in relationship with a girl, her girlfriend falls in love with you. Although you love your girlfriend so much but because her girlfriend gives you money, then, you find yourself turning your attention to her. She buys you shoe polish and other things you need. Finally, if you like to have sex with her, she does not have time to say no. (Iste, 18, rural boy)

For some participants, the effort of a young man to keep one girlfriend may come to an end when his girlfriend refuses to provide sex, then he eventually finds another girl who not only provides it, but also is even ready to financially assist him. Some boys may keep the second girlfriend for financial gain. Money is also mentioned as a limiting factor for boys to engage in concurrent sexual partnerships. Girls want boys to take them out. It is worth mentioning that the majority of young people are unemployed which may limit some young men to entertain current sexual partners.

Most participants view peer pressure as an important factor that encourages sex. Further individual interviews showed that waiting is possible, but peer pressure makes it very hard for young men to have a relationship without sex:

I sometimes think that my classmates and boys we grew up together will look down on me because I do not have sex with my girlfriend. The girlfriend also may laugh at me. Today or tomorrow she is going to say that I was not her boyfriend. Consequently, many friends of mine had sex with girls who have got HIV/AIDS and impregnated them because of such a belief. (Iste, 18, rural boy)

It happens when you are with your friends; they start talking about their girlfriends and how they had sex with them. You are influenced when you find that you are the only one who has never slept with a girl. You may sometimes tell them that you have never slept with a girl, they start giving you strategies to apply for sex. You soon apply them and succeed to have sex with her. After sleeping with her, she may get pregnant but when you tell them what happened, they laugh at you though they are the ones who advised you to sleep with her. Maybe her parents may send you to jail after denying that pregnancy; your friends continue to laugh at you. (Bashengezi, 18, urban boy)

The interviews on this issue brought out the tension of believing one thing as an individual but practising something else because of peer pressure and socially constructed expectations. Boys who experience low self-esteem may rely on others for affirmation. This may lead them to search for self-esteem in sexual encounters. Participants also view peer groups as the training ground where young men discuss sexual persuasive strategies. Despite the encouragement, peers laugh at the boy who impregnates a girl. And if he has a relationship without sexual involvement the girlfriend may also look down on him. From the outset, there is seemingly a good deal of frustration for the boy.

In sum, the findings have shown strong link between both resistance to condom use and concurrent sexual partnerships in association with HIV risk. Participants justified the concurrent sexual partnerships as a way of ensuring that they cannot miss a girl to satisfy their sexual desire. The sexual activity particularly in these conditions may increase an individual’s risk for acquiring HIV.

## Discussion

Qualitative methods were applied to investigate the perspectives of young men about condoms, concurrent sexual partnerships and sex in the context of HIV/AIDS. Results from a series of four focus groups and 20 individual interviews with rural and urban young men suggest that while young men mistrust condoms, they have concurrent sexual partners. If we consider participants’ perspectives, several important points emerged that help understand the reasons why many young men feel reluctant to use condoms.

In response to questions regarding condoms, participants reported that they do not like to use condoms because they are unreliable, ineffective, and have a small hole that is invisible. Consequently, when people use condoms during sexual encounters they may run the risk of contracting sexual diseases. Young men’s stigma about condoms might have increased due to the lack of effective awareness and prevention campaign. Barker and Ricardo (2005) corroborate that young men demonstrate uncertainty and lack of confidence regarding the use of condoms. Condoms are thought to be ineffective or defective. Most participants, in the current study, also believed that condoms can easily break or slip off into vagina during sex. This concurs with a study conducted in Nigeria, in which respondents reported that the inconsistency of condom use is additionally due to widespread distrust of condoms based on respondents’ usage experience, which indicates that ‘condoms burst, tear, and leak’. Consequently, condoms do ‘not offer you 100% protection’ during premarital sex. Respondents associate condom unreliability with both user error and poor product quality (Okonkwo, 2010). In the CADRE’s (2007) survey in South Africa, participants believed that condoms could practically do little to prevent infection. Using condoms entails the possibility of condom failure with the result that a person remains vulnerable as the condom can burst and cause an infection. Crosby, Charnigo, Weathers, Caliendo, and Shrier (2012) and Sanders et al. (2012) find that among the most common errors are condom breakage, slippage, and leakage, which are the primary errors that reduce condom effectiveness. Sanders et al. (2012) argue that the slippage during withdrawal may reflect the user error of not holding the edge of the condom during withdrawal. Some participants in the current study also believed that condoms can cause cancer. Versteeg and Murray (2008), in their study among men and women on condom use in South Africa, found that some participants believed condoms could cause sickness.

The practice of having sex without condoms was also motivated by males’ belief that having sex with condoms can diminish pleasure during sexual intercourse. Therefore, most participants subscribed to beliefs such as ‘you cannot eat candy in a wrapper’, which encourages them to deliberately engage in unprotected sex (‘flesh-to-flesh’) as a sign of celebrating their maleness. They reiterated the desire to have ‘flesh-to-flesh’ sex, i.e. sex without a condom. These attitudes and their impact on risk-taking behaviour may be linked to the spread of HIV. Participants therefore, indicated they wanted ‘flesh-to-flesh’ or ‘skin-to-skin’ sex. They also mentioned that the commercialisation and distribution of condoms encourage sexual promiscuity in the community. A number of studies in South Africa have discussed the above assumption. Versteeg and Murray (2008) indicate that participants believed condoms decrease sexual pleasure and hinder men to perform positively during the sexual act. Ackermann and De Klerk (2002) find that men mentioned that condoms decrease sexual gratification. Hunter (2002) demonstrates, in his study conducted in KwaZulu-Natal, that dominant masculinity sometimes shapes men's violent control over women, and encourages men to insist on ‘flesh to flesh’ sex. He also notes that men convince women that condom use leads to ‘bad sex’, and is associated to ‘unfaithful’ behaviour. They believe that true love is represented by *inyama enyameni* (‘flesh-to-flesh’ sex). MacPhail and Campbell (2001) claim that the concept of masculinity is connected to the belief of unprotected sex, ‘flesh to flesh’, as being more pleasurable. Hunter (2002), and Wood, Lambert, and Jewkes (2008), indicate that in South Africa dominant masculinities construct men’s excessive control over women and their insistence on having ‘flesh to flesh’ sex. This also corroborates the findings of Njue, Voeten, and Remes (2011) in Kenya, in which young men mentioned trust, discomfort and the reduction of pleasure as reasons for not using condoms. For most girls, not choosing to use a condom was connected to their limitation to demand its use, intimacy and pleasure, and mistrust about its safety. Sanders et al. (2012), in their study in the United States, maintain that the ineffectiveness of condoms may not only compromise condom effectiveness, but may also discourage condom use if people become frustrated or have less pleasurable experiences as a result of their use. However, Crosby, Graham, Yarber, and Sanders (2010) argue that problems with erection reduced pleasure (for both partners) and failure of the female partner to orgasm were noticed more frequently among men reporting a lack of time for condom application.

The girl’s request to use a condom was viewed with distrust. A number of participants commented that if their girlfriend or (future) wife asked them to use a condom, they would interpret it as a lack of trust in them. They would also suspect her of being unfaithful. Women who suggest condoms are viewed as carriers of disease. The girl’s request to use a condom is also seen as a lack of love towards the male partner. Participants perceived certain threats to reputations of women suggesting condoms, especially when she demonstrates having the skill and confidence to negotiate condom use that implies sexually activity and experience in initiating sex, with potentially negative associations. Women are thus unable to insist on condom use due to the lack of communication regarding sexual matters and power in interpersonal relationships. These inequalities may reduce female voice in sexual negotiation. In such situation, the possibilities for women to insist on condoms are very limited. Therefore, having sex and the control around it only depends on the men’s will. Most of the participants’ perceptions clearly described how the imbalance of gender power impedes women’s power to negotiate safe sex. Gender power disparity significantly hinders their ability to negotiate and suggest condoms, and further exposes both partners to Sexually Transmitted Infections and HIV. Ackermann and De Klerk (2002), in their study in South Africa, indicated that if a woman suggests the use of condom, men accuse of being unfaithful or hiding sexually transmitted diseases. This also corroborates with Hallman’s (2004) study conducted among young women and men aged 14–24 years in KwaZulu-Natal, which finds that young men consider women who suggest condom use as loose. The simple talk about condoms use could lead to psychological, corporal and economic abuse. Various studies conducted in sub-Saharan Africa found that sex among adolescents often results in very little negotiation or communication. Condom use is the mostly difficult point among youth since it refers to uncleanliness and infidelity, and many girls describe a fear of punishment for initiating a conversation on this issue (Varga, 1997). MacPhail and Campbell’s (2001) study conducted in South Africa, also finds that these sexual behaviours are reinforcing among peers. Young men are criticised for using a condom and, as a result, may decide to not use it again. A study conducted in Kinshasa and Bukavu finds that condom use was related to promiscuity rather than being seen as an act of accountable behaviour based on respect for the adolescent’s own well-being and that of his or her girlfriend or boyfriend (Bosmans et al., 2006). Langen (2005) observed similar patterns in her research in South Africa and Botswana, where gender power disparity significantly reduces women’s power to suggest condoms to their boyfriends.

Some participants mentioned that they are reluctant to use condoms because condoms are man-made, and the Bible forbids men to waste sperm. This indicates that religious thinking is one of important factors behind young men’s beliefs on condom use. This corroborates Muanda et al.’s (2017) study conducted in other three provinces of the DRC in which some participants reported such beliefs. Bosmans et al. (2006) confirm that sociocultural barriers to discuss sexuality and the promotion of condom use were reinforced by strict obedience to Catholic doctrine. Religion was used as the justification for reducing the ‘ABC’ approach (abstinence, be faithful, condom use), whereby abstinence was promoted among adolescents as the only responsible way of behaving. The Refugee Council (2004) argues that in the DRC, contraception is not often used, particularly in rural areas, and that some religious leaders are opposed to it because they believe that it will encourage immorality. Brummer (2002) maintains that condoms were seen as ‘not natural’ or people doubted of their ability to guaranteeing safe sex. However, a few participants, in the current study, advised young men to leave religious beliefs aside, and only focus on how to prevent themselves from the risk of contracting sexually transmitted diseases.

It is clear that the majority of participants deliberately ignored the risk of contracting Sexual Transmitted Infections, and have strong inclination to concurrent sexual partnerships and inconsistent condom use. Most participants actually justified the concurrent sexual partnerships as a way of ensuring that they cannot miss a girl to satisfy their sexual desire. Some participants saw the benefit in having one girl for a trusting relationship and another essentially for sex. Concurrent sexual partnerships may encourage the spread of Sexually Transmitted Infections especially in the context of South Kivu as men reject condom use. CADRE (2007), in a South African cross-sectional survey, found that concurrent sexual partnerships give hope and provide a supportive structure that ensures that sensitive support is always available. In particular, having another partner provides a cushion of support, should a current relationship end. Wood et al. (2008), in their study conducted in South Africa among young people, found that it was common for young people to have more than one partnership at a time whether casual or more serious (and an entire slang terminology was used to describe sexual partners positioned differentially in the hierarchy of an individual’s relationships). Rakgoasi (2010), in his study in Botswana, found that concurrent sexual partnerships were necessary if partners refuse to have sex with them, especially if the reasons for their partner’s reluctance towards sex are not clear to them. To many men, they then engage in concurrent sexual partnerships, which are needed to meet their need for sex; two girlfriends are enough, largely because of financial commitments. Participants in the current study also reported that peer pressure makes it very hard for them to have a relationship without sex. They implied that a relationship between a man and a woman must involve sex so that when this ends she cannot consider him useless or not enough of a man, and thus bad mouth him when she is among her peers. An ethnographic study conducted in North Western Tanzania describes how one of the participants had a long relationship with his girlfriend, which only involved talking, joking and caressing, until his male friends encouraged him to have sex with her (Wamoyi, Wight, Plummer, Mshana, & Ross, 2010). The study conducted in Kenya by Njue et al. (2011) finds that peer influence was a great driving force for risky sexual behaviours. Wood et al. (2008) demonstrate how in South Africa the control of women became an important aspect of successful masculinity among young men, which is proved through their ability to have the most desirable girlfriends, and to control their girlfriends. Masculinity was constructed as an on-going challenge among male peers. Sexual conquest was considered a sign of status mostly attained by wooing, trickery and the use of force. Barker and Ricardo (2005) argue that men often aim to demonstrate their masculinity before their male friends and social group within restricted ideas of what it means to be a man. They describe a sense of being monitored and watched to see if they meet the standard versions of masculinity. Other men and women assess the achievement of masculinity.

Most participants viewed concurrent sexual partnerships as an opportunity to make a choice of spouse from among their girlfriends. The practice may assist them in making a better choice in terms of marriage. They stated that they need to maintain concurrent sexual partners because of the idea that having concurrent sexual partners would allow them to compare and select the partner who is most suitable for marriage. Character is seen as a more reliable criterion for selecting wives. Participants believed that a girlfriend’s appearance and character determine trustworthiness. Women who reject the sexual advances of other men, and who are viewed as ‘controlled’, gain value as the best future spouses. Although participants indicated the importance of concurrent sexual partners, when they want to marry, they do not marry girls with whom they have had sex. They mentioned that they choose their future spouses on the basis of their [girls’] reputation. This may result in the girl’s hypocrisy. As a reason for responding negatively to requests for sex, girls often say no although they may desire it, they are afraid their boyfriends will afterwards despise them, consider them prostitutes, and recount their sexual endeavours to their peers (Mulumeoderhwa & Harris, 2014). A study in South Africa reports that participants support concurrent sexual partners as a way of ensuring that men cannot miss a girlfriend if one ‘breaks your heart’, and this practice also helps men make the choice of a future spouse (Mulumeoderhwa & Harris, 2013).

## Limitations of the study

Considering that this study took place among 28 students in four schools in one province of the DRC, it would be inappropriate to generalise its findings. Young women were not included in the study which weakens the paper; we admit that their voice would have made the paper much stronger. In the future, other researchers need to investigate the girls’ attitudes on condoms and concurrent sexual partners, and also the role that churches play in influencing the condom use. It can be hoped that those concerned with reducing gender inequality in the DRC and elsewhere will find the research insightful.

## Recommendations

Given the boys’ failure to use condoms and their strong inclination to concurrent sexual partnerships, there is a need for heath groups and stakeholders within the area to increase awareness about condoms’ effectiveness and improve knowledge dissemination on Sexually Transmitted Diseases and how they are prevented. However, this is not the place to discuss how such attitudes could be cost effectively spread; that would require a separate study. Peacock and Levack (2004), Chege (2005) and Petersen, Bhana, and Mckay (2005), among others, have reviewed various efforts of ‘constructive male involvement’ to reduce levels of gender violence. These findings also underscore the need for prevention efforts targeting fidelity which may foster sexual behaviour change, and ultimately curb the spread of HIV.

## Conclusion

This study has shown up that the strong mistrust of condoms and strong support for concurrent sexual partnerships may hinder the effectiveness of the fight against HIV/AIDS. The study has also found that negotiation regarding condom use is missing in many sexual relationships involving young people in South Kivu, and condom use remains unpopular in male-female relationships. Most participants had strong reservations on condoms and were positively inclined to the notion of having concurrent sexual partnerships. The study has also demonstrated that the imbalance in power relations between male and female partners in heterosexual relationships may hold a grip over the ability of young women to either refuse sex or negotiate the use of condoms. Thus, the boys’ failure to use condoms and their strong inclination to concurrent sexual partnerships can significantly increase the spread of Sexually Transmitted Infections and HIV.
